# Saturated Fatty Acid-Induced Cytotoxicity in Liver Cells Does Not Involve Phosphatase and Tensin Homologue Deleted on Chromosome 10

**DOI:** 10.1155/2013/514206

**Published:** 2013-04-15

**Authors:** Dong Wang, Yuren Wei, Melinda Frye, Christopher L. Gentile, Michael J. Pagliassotti

**Affiliations:** ^1^Department of Food Science and Human Nutrition, Colorado State University, 234 Gifford, Fort Collins, CO 80523-1571, USA; ^2^Department of Biomedical Sciences, Colorado State University, Fort Collins, CO 80523, USA

## Abstract

Liver specific deletion of the tumor suppressor phosphatase and tensin homologue deleted on chromosome 10 (PTEN) induces steatosis and hypersensitivity to insulin. Saturated fatty acids, which induce endoplasmic reticulum stress and cell death, appear to increase PTEN, whereas unsaturated fatty acids which do not induce endoplasmic reticulum stress or cell death reduce this protein. In the present study, the role of PTEN in saturated fatty acid-induced cytotoxicity was examined in H4IIE and HepG2 liver cells. Palmitate and stearate increased the expression of PTEN, whereas the unsaturated fatty acids, oleate and linoleate, reduced PTEN expression in both cell types. SiRNA-mediated knockdown of PTEN did not increase liver cell triglyceride stores or reduce palmitate- or stearate-mediated ER stress or apoptosis. These results suggest that PTEN does not play a significant role in saturated fatty acid-induced cytotoxicity in these liver cell models and in the absence of insulin.

## 1. Introduction

Nonalcoholic fatty liver disease (NAFLD) is a chronic metabolic disorder characterized by an increase in the hepatic triglyceride pool (steatosis) and, in some individuals, development of nonalcoholic steatohepatitis (NASH) [1]. Accumulating data suggest that, in addition to their role in the development of hepatic steatosis [2], fatty acids play an important role in disease progression. Free fatty acids are elevated in patients with NASH and are positively correlated with disease severity [3, 4]. Suppression of circulating fatty acids improved hepatic insulin sensitivity and reduced liver enzymes in healthy individuals [5]. Data such as these have led to the emerging concept that elevated fatty acids and products of fatty acid metabolism, rather than the triglyceride pool per se, promote hepatotoxicity and disease progression in NAFLD. Indeed, hepatic triglycerides are higher in patients with benign steatosis compared to those with NASH [6], and stimulation of lipogenesis and/or fatty acid oxidation protects against palmitate-induced cell death in *β*-cells [7]. 

The cytotoxic response to elevated fatty acids appears to be specific to or made more severe by long-chain saturated fatty acids [8–11]. In hepatocytes, saturated fatty acids promote endoplasmic reticulum (ER) stress and apoptosis [11, 12], both of which are characteristic features of NAFLD [13, 14]. The toxic effects of saturated fatty acids may be due to their inability, relative to unsaturated fatty acids, to be esterified and incorporated into triglyceride [7, 15–17]. Lipid phosphatase and tensin homolog deleted on chromosome 10 (PTEN) is a multifunctional phosphatase that converts phosphatidylinositol-3,4,5-triphosphate (PIP_3_) to phosphatidylinositol bisphosphate [18]. One function of PTEN is to antagonize insulin signaling by reducing the level of PIP_3_ [19]. PTEN also appears to be involved in the development of hepatic steatosis and regulation of hepatic triglyceride stores [20]. Liver specific deletion of PTEN resulted in hepatic steatosis in mice [19] and PTEN expression was reduced in the liver of rats with steatosis and high plasma levels of fatty acids, as well as in human livers characterized by steatosis [20]. In addition, unsaturated fatty acids, which are readily incorporated into hepatocyte triglycerides, reduce the expression of PTEN in HepG2 cells [20, 21]. Therefore, the aim of this study was to examine the role of PTEN in saturated fatty acid-mediated ER stress and apoptosis in liver cells. We hypothesized that saturated fatty acid-mediated ER stress and apoptosis involved increased expression of PTEN. 

## 2. Materials and Methods

### 2.1. Cell Culture

The rat hepatoma liver cell line H4IIE and the human hepatoma cell line HepG2 (American Type Culture Collection, Manassas, VA, USA) were cultured in DMEM containing 8 mM glucose and supplemented with 10% fetal bovine serum, penicillin, and streptomycin sulfate. Experiments were performed in the absence (LG) or presence of fatty acids that were complexed to bovine serum albumin at a 2 : 1 molar ratio [11] and were 16 h in duration. Each experiment was performed in triplicate and a total of 6 independent experiments were performed for each cell line and treatment.

### 2.2. Transfection

PTEN SiRNA or scrambled SiRNA (50 nM; Cell Signaling, Waverly, MA, USA) was transfected into H4IIE or HepG2 liver cells using TransIT-siQuest transfection reagent (Mirus Bio, Madison, WI, USA). Experiments were performed 24 h following transfection. 

### 2.3. Triglyceride Analysis

Total lipids were extracted using the methods of Bligh and Dyer [22]. Triglyceride concentration was determined using a kit (Zen-Bio, Research Triangle Park, NC, USA). 

### 2.4. RNA Isolation and Analysis

Total RNA was extracted with Trizol reagent using the manufacturer's protocol (Invitrogen, Carlsbad, CA, USA). Real Time PCR was performed following reverse transcription using 0.5 *μ*g of DNAase-treated RNA, Superscript II RnaseH, and random hexamers. PCR reactions were performed using transcribed cDNA and IQ-SYBR green master mix (Bio Rad, Hecula, CA, USA) as described in detail previously [11]. Analysis of spliced XBP1 was based on previously described methods [23] using the following primers: Forward: 5′GTCTGCTGAGTCCGCAGCAGG3′ and Reverse: 5′GATTAGCAGACTCTGGGGAAG3′. 

### 2.5. Immunoblot Analysis

Cell processing and immunoblot analysis were performed as described previously [11]. Primary antibodies included total and phosphorylated eukaryotic initiation factor2*α* (eIF2*α*, Cell Signaling) and glucose-regulated protein-78 (GRP78, Santa Cruz Biotechnology, Santa Cruz, CA, USA). Proteins were detected using horseradish peroxidase-conjugated secondary antibodies and density was determined using a UVP Bioimaging system (Upland, CA, USA). 

### 2.6. Analysis of Akt

Total and phosphorylated (serine 473) Akt was determined using the STAR (Signal Transduction Assay Reaction) ELISA kit (Millipore) per the manufacturer's instructions. This kit is based on a solid phase sandwich enzyme-linked immunosorbent assay in which 96-well plates are coated with a monoclonal Akt antibody, and following incubation with cell lysates Akt is detected using a specific rabbit anti-Akt1 antibody or phosphorylated Akt is detected using a specific rabbit anti-phospho-Akt (Ser473) antibody. 

### 2.7. Cell Viability

Cell death was evaluated using the Cell Death Detection ELISA kit (Roche Diagnostics, Penzberg, Germany). This assay is based on the quantitative sandwich-enzyme-immunoassay principle using mouse monoclonal antibodies directed against DNA and histones. Cell survival was evaluated using 3-(4,5-dimethylthiazol-2-yl)-2,5-diphenyltetrazolium bromide (MTT) assays (Promega Inc., Madison, WI, USA) based on the supplier's protocol. Caspase-3 activity was determined with the Colorimetric Caspase-3 Activation Assay, which uses a caspase specific peptide conjugated to the color reporter *p*-nitroaniline (R&D Systems, Minneapolis, MN, USA). 

### 2.8. Statistics

Statistical comparisons were made based on a factorial design using 2-way ANOVA with Bonferroni's post hoc test or 1-way ANOVA with the least significant difference test. The level of significance was *P* < 0.05. Data are reported as means ± standard deviations for 6 independent experiments in each cell type.

## 3. Results

### 3.1. Individual Fatty Acids Differentially Influence PTEN Expression

**Figure 1:**
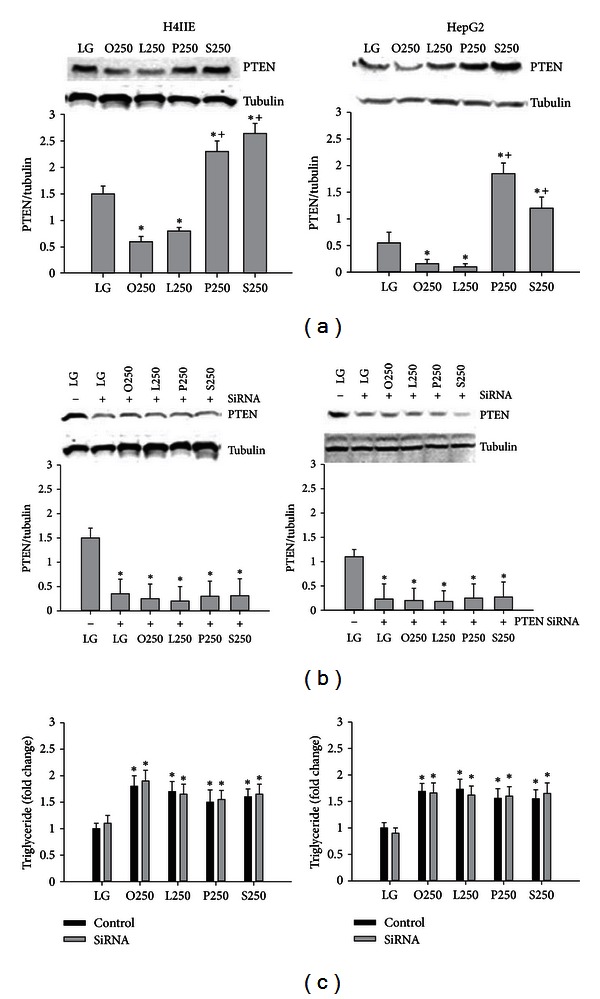
PTEN expression and effects of PTEN knockdown on triglyceride stores in liver cells. PTEN protein expression following 16 h of incubations with control media (LG) or fatty acids (oleate (O), linoleate (L), palmitate (P), or stearate (S)) at 250 *μ*M (a) in H4IIE (left column) or HepG2 (right column) liver cells (*n* = 6). Effectiveness of SiRNA-mediated knockdown of PTEN in liver cells (b). Effects of SiRNA-mediated knockdown of PTEN on triglyceride stores in liver cells following 16 h of incubations with control media or fatty acids (c). *: significantly (*P* < 0.05) different from LG; ^+^: significantly (*P* < 0.05) different from O250 and L250.

The unsaturated fatty acids, oleate and linoleate, reduced PTEN expression, whereas the saturated fatty acids, palmitate and stearate, increased PTEN expression in both H4IIE and HepG2 liver cells (Figure 1(a)). 

### 3.2. SiRNA-Mediated Knockdown of PTEN Does Not Increase Triglyceride Stores or Mitigate Saturated Fatty Acid-Induced ER Stress and Cell Death

**Figure 2:**
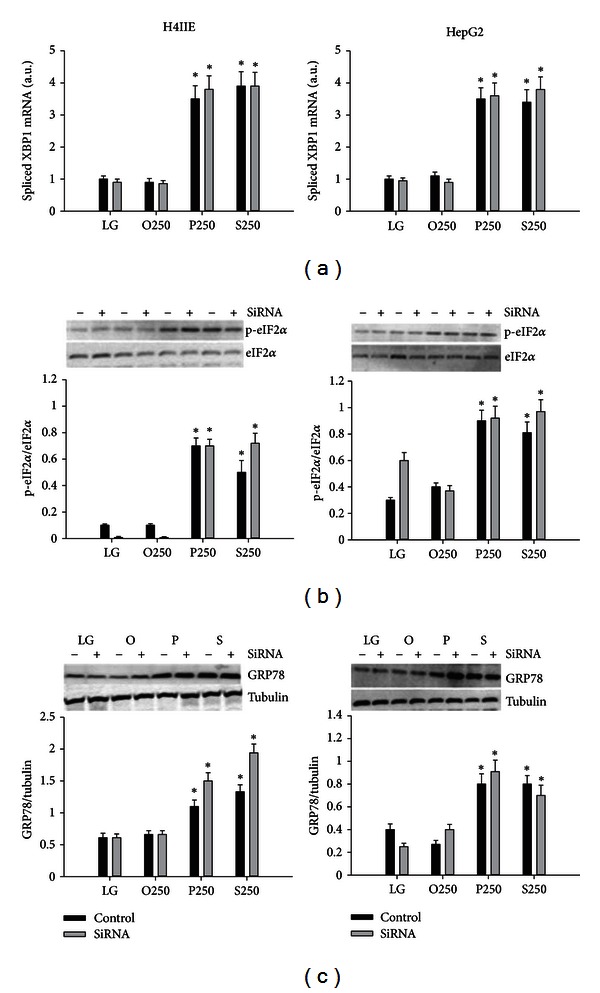
ER stress markers in H4IIE and HepG2 liver cells. XBP1 mRNA splicing (a), phosphorylation of eIF2*α* (b), and GRP78 protein expression (c) in H4IIE (left column) and HepG2 (right column) liver cells following 16 h of incubations with control media (LG) or fatty acids (oleate (O), linoleate (L), palmitate (P), or stearate (S)) at 250 *μ*M in the absence (Control) or presence of PTEN knockdown (SiRNA) (*n* = 6). *: significantly (*P* < 0.05) different from LG and O250.

**Figure 3:**
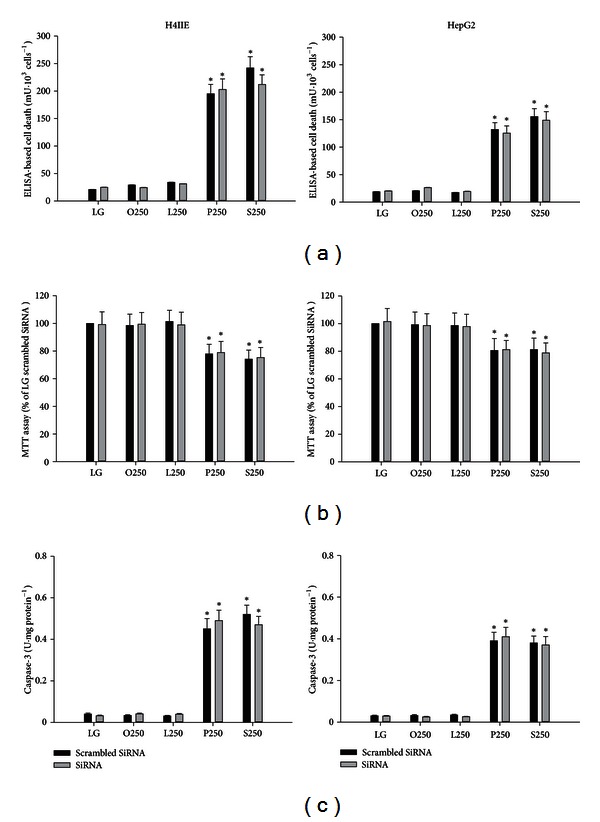
Cell viability in H4IIE and HepG2 liver cells. ELISA-based cell death (a), MTT assay (b), and caspase-3 activity (c) in H4IIE (left column) and HepG2 (right column) liver cells following 16 h of incubations with control media (LG) or fatty acids (oleate (O), linoleate (L), palmitate (P), or stearate (S)) at 250 *μ*M in the absence (Control) or presence of PTEN knockdown (SiRNA) (*n* = 6). *: significantly (*P* < 0.05) different from LG, O250, and L250.

Previous studies demonstrated that liver specific PTEN deletion increased hepatic steatosis; therefore we hypothesized that increased expression of PTEN may account for saturated fatty acid-mediated cytotoxicity by restricting the incorporation of these fatty acids into the triglyceride pool. SiRNA for PTEN resulted in a 75% reduction in the expression of PTEN (Figure 1(b)) and prevented the saturated fatty acid-induced increase in PTEN (data not shown). However, PTEN knockdown did not increase triglyceride concentrations in response to oleate, linoleate, palmitate, or stearate in H4IIE or HepG2 liver cells (Figure 1(c)). PTEN knockdown did not reduce markers of ER stress (Figure 2) or cell death (Figure 3) in response to palmitate or stearate. 

### 3.3. SiRNA-Mediated Knockdown of PTEN Increases Basal and Insulin-Stimulated Phosphorylation of Akt

**Figure 4:**
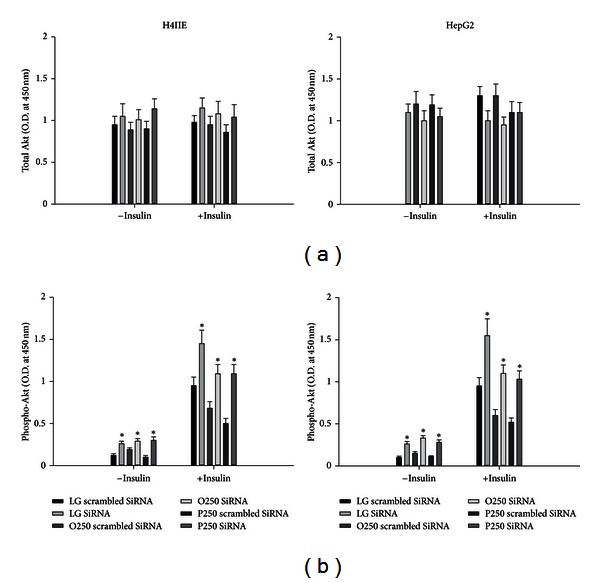
Effects of PTEN knockdown on phosphorylation of Akt in liver cells. Total Akt (a) and phosphorylated Akt (b)) in H4IIE (left column) and HepG2 (right column) liver cells following 16 h of incubations with control media (LG) or fatty acids (oleate (O) or palmitate (P)) at 250 *μ*M. Following incubations, cells were either treated (+ insulin) or not treated (− insulin) with 1 nM insulin for 30 minutes (*n* = 6). *: significantly (*P* < 0.05) different from corresponding scrambled SiRNA group.

 To examine whether the inability of PTEN knockdown to influence triglyceride stores or saturated fatty acid-induced ER stress and cell death was due to a general ineffectiveness of the knockdown, we examined phosphorylation of Akt. PTEN knockdown increased the phosphorylation of Akt in the absence and presence of insulin (Figure 4). These results suggest that our observations with respect to PTEN in the context of saturated fatty acid-induced ER stress and cell death were not due to a general ineffectiveness of the knockdown. 

## 4. Discussion

Lipid phosphatase and tensin homologue deleted on chromosome 10 (PTEN) converts phosphatidylinositol-3,4,5-triphosphate (PIP_3_) to phosphatidylinositol bisphosphate. Thus, PTEN antagonizes insulin signaling in the liver by reducing the levels of PIP_3_. Hepatocyte specific deletion of PTEN results in accelerated lipogenesis and hepatic steatosis. A previous study demonstrated that the unsaturated fatty acids, oleate and palmitoleate, but not saturated fatty acids reduced the expression of PTEN. We hypothesized that the inability of saturated fatty acids to downregulate PTEN might restrict diversion into the triglyceride pool and therefore result in ER stress and cell death. We first examined the effects of unsaturated and saturated fatty acids on PTEN expression. The unsaturated fatty acids, oleate and linoleate, reduced PTEN, whereas the saturated fatty acids, palmitate and stearate, increased PTEN expression in H4IIE and HepG2 liver cells. However, knockdown of PTEN did not result in an increase of triglyceride stores nor did it reduce ER stress or cell death in response to palmitate or stearate. These data suggest that PTEN expression is not a critical determinant of saturated fatty acid-mediated ER stress or cell death in these liver cell models. 

High plasma free fatty acid and triglyceride concentrations can lead to increased import of free fatty acids into both adipose tissue and nonadipose tissues, such as liver, heart, and pancreas, and lead to intracellular lipid accumulation [24]. The accumulation of triglyceride in liver cells is a characteristic feature of nonalcoholic fatty liver disease and has been proposed as an initiating event in disease progression [1]. Accumulating data suggest that, in addition to their role in the development of hepatic steatosis [2], fatty acids play an important role in disease progression. Free fatty acids are elevated in patients with NASH and are positively correlated with disease severity [3, 4]. Suppression of circulating fatty acids improved hepatic insulin sensitivity and reduced liver enzymes in healthy individuals [5]. Data such as these have led to the emerging concept that elevated fatty acids and products of fatty acid metabolism, rather than the triglyceride pool per se, promote hepatotoxicity and disease progression in NAFLD. A number of recent in vitro and in vivo studies have demonstrated that the different forms of free fatty acids exert remarkably different effects. Exposure of a variety of cell types, including hepatocytes, to long-chain saturated fatty acids led to increased inflammation, ER stress, and cell death [11, 15, 25]. It has been proposed that a key event determining saturated fatty acid-induced toxicity involves its reduced ability to form triglyceride [15, 26]. In this paper we hypothesized that the differential effects of saturated fatty acids on PTEN expression mediated saturated fatty acid-induced toxicity. However, the results do not support this hypothesis. 

The present series of experiments were performed in the absence of insulin and as a result have likely minimized the impact of PTEN expression on saturated fatty acid-mediated cytotoxicity. PTEN is an important regulator of insulin signaling, and recent studies have demonstrated that the ability of PTEN to regulate hepatic lipid synthesis and storage is mediated via these effects and in concert with Akt2 [27]. Thus, we cannot rule out the possibility that PTEN may contribute to saturated fatty acid-mediated cytotoxicity under conditions in which insulin is present. The present series of experiments do suggest that saturated fatty acid-mediated changes in PTEN expression do not restrict expansion of the triglyceride pool nor do they account for the subsequent induction of ER stress and apoptosis. 

In the present study, the role of PTEN in saturated fatty acid-induced cytotoxicity was examined in H4IIE and HepG2 liver cells. Palmitate and stearate increased the expression of PTEN, whereas the unsaturated fatty acids, oleate and linoleate, reduced PTEN expression in both cell types. SiRNA-mediated knockdown of PTEN did not increase liver cell triglyceride stores or reduce palmitate- or stearate-mediated ER stress or cell deaths.
